# Function diversity of the expansin subfamily genes in *Populus tomentosa*

**DOI:** 10.3389/fpls.2025.1741986

**Published:** 2026-01-22

**Authors:** Junkang Zhang, Han Zhao, Hao Zhang, Mengjia Yang, Yuxi Chen, Jichen Xu

**Affiliations:** State Key Laboratory of Tree Genetics and Breeding, College of Biological Sciences and Technology, Beijing Forestry University, Beijing, China

**Keywords:** diversity, expansin, growth, populus tomentosa, resistance, subfamily

## Abstract

Expansins are crucial components in plant cell walls and are classified into four subfamilies based on their sequences. To investigate their function differentiation in plant growth and stress resistance, four expansin subfamily genes—*PtoEXPA8*, *PtoEXPB3*, *PtoEXLA2*, and *PtoEXLB1*—were cloned from *Populus tomentosa* Carr. TC1521. Their sequence and structure exhibited typical subfamily characteristics. They were individually introduced in tobacco plants and made different contributions to the plants’ performances. Compared to wild-type plants, the *PtoEXPA8* overexpressing lines increased the leaf area, and *PtoEXPB3* facilitated floral organ development and earlier flowering and increased flower diameter. *PtoEXLA2* increased plant height but reduced flower diameter and 1000-seeds weight. Finally, *PtoEXLB1* increased plant height and decreased flower diameter. Under heat stress conditions, compared to the wild-type plants, the *PtoEXPA8* overexpressing plants performed the best, while the other three genes barely contributed to heat resistance. The results indicate that the expansin subfamily genes underwent significant function differentiation, playing distinct roles in plant growth and stress resistance in poplar.

## Introduction

Expansins are cell wall components that disrupt the non-covalent bonds between cellulose, xyloglucan, and hemicellulose, thereby increase the flexibility of cell walls and relieve tension (McQueen-Mason et al., 1992). Plant genomes contain dozens of expansin genes, which are divided into four subfamilies based on their sequence homology: *EXPA, EXPB, EXLA*, and *EXLB*. In plant species, the expansin family composition varies significantly, such as 33 *EXPA*, 18 *EXPB*, 4 *EXLA*, and 1 *EXLB* genes in rice (Sampedro and Cosgrove, 2005) and 25 *EXPA*, 3 *EXPB*, 1 *EXLA*, and 2 *EXLB* genes in rose (Zheng et al., 2024). Each gene features a specific structural attribute based on its subfamily: the *EXPA* subfamily genes generally have introns of types A and B, while other subfamilies possess all the four intron types of A, C, B, and F. Only the *EXPA* and *EXPB* subfamily genes possess the conserved HFD motif (Cosgrove, 2015).

Some expansin genes are crucial for plant growth and development: *AtEXPA2* and *BdEXPA27* for seed germination (Sanchez-Montesino et al., 2019; Chen et al., 2020), *ZmEXPB6* and *NtEXPA11* for plant leaf development (Geilfus et al., 2015; Marowa et al., 2020), *AcEXPA23, GmEXLB1*, and *HvEXPB7* for root development (Wu et al., 2022; Kong et al., 2019; He et al., 2015), *PbrEXLA3* for flower development (Wang et al., 2023b), and *MdEXLB1* and *SlExp1* for fruit ripening (Chen et al., 2022; Su et al., 2024). Some expansin genes were also associate with stress resistance: *AsEXP1* and *PttEXPA8*, for instance, were found to contribute to heat resistance in C3 Agrostis grass and *Populus tomentosa* (Xu et al., 2007; Liu et al., 2016); *OfEXLA1* and *EaEXPA1* facilitate drought resistance in *Osmanthus fragrans* and *Erianthus arundinaceus* (Dong et al., 2024; Ashwin et al., 2021); *TaEXPA8, TaEXPB7-B, AnEXPA1*, and *AnEXPA2* are linked to resistance to cold stress in *Triticum aestivum* and *Ammopiptanthus nanus* (Peng et al., 2019; Feng et al., 2019; Liu et al., 2019); *ZmEXPB2, ZmEXPB6, ZmEXPB8, SmEXPA13*, and *CqEXPA50* contribute to salt resistance in *Zea mays*, *Salix matsudana*, and *Chenopodium quinoa* (Geilfus et al., 2010, 2015; Zhang et al., 2023; Sun et al., 2022b); *SbEXPA11* is related to cadmium stress resistance in sorghum (Wang et al., 2023a); and *OsEXPA1, OsEXPA5, OsEXPA10, OsEXPB7*, and *MaEXPA11* help disease resistance in *Oryza sativa* and *Fructus mori* (Ding et al., 2008; Guo et al., 2024). Evidently, the expansin genes demonstrate a certain function differentiation.

Poplar, a model species of woody plants, is also an important tree for landscaping and industrial timber. A Total of 36 expansin genes were identified in the poplar genome: 27 *EXPA* genes, 3 *EXPB* genes, 2 *EXLA* genes, and 4 *EXLB* genes (Li et al., 2014). To understand how their functions differed, four subfamily expansin genes were cloned from *Populus tomentosa* TC1521, and sequence analysis was performed to discover their subfamily characteristics. The genes were then introduced in tobacco plants, and their contributions to plant growth and stress resistance were tested. The results help us understand the significance of the expansin family and offer a basis for molecular design breeding in forest trees.

## Materials and methods

### Plant materials and treatment

The cuttings of *Populus tomentosa* Carr. TC1521 were planted in the pots (10 cm × 10 cm) filled with vermiculite, and grew in the growth chamber (2000 lux light intensity, 16/8 h light/dark cycle, 25°С). The plants were irrigated with water every three days, and applied with Hoagland nutrient solution once a week. After growing for three months, the plants were subjected to a 42 °C treatment for 3 d (25 °C as control). The total RNA was extracted from the matured leaves for gene cloning and physiological index tests (see next).

### Cloning and expression analysis of the poplar expansin genes

The total RNA was extracted from the leave samples using the Trizol method (Invitrogen, USA) and was then reverse transcribed into cDNA. The four genes were selected from each expansin subfamily of *Populus tomentosa* genome: *PtoEXPA8, PtoEXPB3, PtoEXLA2*, and *PtoEXLB1*. The specific primers for each gene were designed based on the poplar genome data (http://www.phytozome.net/poplar) (**Supporting Information Table S1**). The expression patterns of the four genes were detected using the poplar cDNA samples with *UBQ* as the reference. The PCR was conducted in 35 cycles at 94 °C for 30 s, 55 °C for 30 s, and 72 °C for 1 min. Three plants were tested as replicates. The values significant difference between the control (25°C) and heat treatment (42°C) (P<0.01) was determined by student’s t-test.

### Gene transformation into tobacco plants

The specific primers were designed at both side of the expansin gene with HindIII and XbaI restriction enzyme sites (**Supporting Information Table S2**). The full-length gene fragment was amplified from the cDNA sample under heat treatment, and ligated to the T-blunt cloning vector. The cloned gene fragment and plant expression plasmid pEZR(K)-LC were digested with HindIII and XbaI and then ligated with T4 ligase. The recombinant plasmid was introduced into *Agrobacterium tumefaciens* strain GV3101 by electroporation, and the positive strain was determined by PCR test. The activated agrobacterium strain was inoculated to YEB liquid medium, and cultured at 28 °C for 7–8 h until until OD_600_ up to 0.5-0.6. The healthy tobacco leaves were cut and immersed in the bacterial solution for 10 min, then transferred on the screening medium (MS + 2 mg/L 6-BA + 0.2 mg/L NAA + 3% sucrose + 0.55% agar) in dark for 3 d, and further on the differentiation medium (MS + 2 mg/L 6-BA + 0.2 mg/L NAA + 3% sucrose + 0.55% agar + 100 mg/L Kana + 200 mg/L Timentin) in light. The regenerated buds were transferred on the rooting medium (MS + 3% sucrose + 0.55% agar + 50 mg/L Kana + 200 mg/L Timentin). DNA was extracted from the seedlings and used for PCR tests by the specific primers. RNA was extracted from the positive lines based on DNA test, retrotranscribed and assayed for the gene expression by PCR. There transgenic lines with similar expression level for each gene were used for the evaluation of the subfamily gene effect on plant growth and development and stress resistance.

### Evaluation of the tobacco plants in growth and physiological indexe*s*

The transgenic plants were planted. The F1 seeds were harvested, planted, and had DNA-tests. The F2 seeds were harvested from each positive F1 line, planted, and had DNA-tests again (20 seedlings for each line). The all F2 plants from a single F1 plant having the transformed gene were recognized as homozygous. Their F3 seeds were harvested and used for the next assays. The F3 transgenic tobacco lines and wild types (WT) were planted in pots (10 cm × 10 cm) with vermiculite and grew in the growth chamber. The plants were irrigated with water every 3 d and Hoagland solution once a week. Three identical plants of each line were used for the measurement of the growth indexes (Wang et al., 2011). The plant height was from the base to the top of the plants in the maturity stage. The flower diameter and receptacle length were assessed at the full bloom period. A total of 1,000 plump seeds were weighed to obtain the thousand-grain weight value. The fifth leaf from the top of the mature plants was harvested and measured for the leaf area using a YMJ-A leaf area meter.

The one-month old tobacco seedlings were treated at 42 °C for 3 d (25 °C as control). The leaves were harvested for physiological index tests. Three replicates for each line/treatment were conducted. SPSS23.0 software was used for multiple comparison among lines at significant difference level p = 0.05.

Relative Electrical Leakage (REL): 0.1 g fresh leaf sample was cut into small pieces (0.5 cm × 0.5 cm) and immersed in 30 mL of deionized water, then shaken at 180 rpm for 1 d. The electrical conductivity was measured as R1. The samples were further autoclaved at 121 °C for 20 min and shaken for 24 h again, and the electrical conductivity was measured as R2 (Zhang et al., 2018). The REL was calculated by the formula: REL (%) = (R1/R2) × 100%.

Malondialdehyde (MDA) content: 0.1 g fresh leaf sample was ground with 1 mL of 10% TCA to form a homogenate, then centrifugated at 12,000 rpm for 10 minutes. The supernatant was mixed with equal volume of 0.6% TBA solution, subjected to boiling water bath for 15 minutes, then centrifugated at 12,000 rpm for 10 minutes. The supernatant was used for the absorbance values measurement at wavelength of 450 nm, 600 nm, and 532 nm, respectively (Wang et al., 2018). The MDA content was calculated by [6.45 × (OD_532_ - OD_600_) - 0.56 × OD_450_] × total extract volume/fresh weight of sample.

Chlorophyll content: the fifth leaf of the plants was selected for chlorophyll content measurement by chlorophyll meter SPAD-502Plus (Konica Minolta Sensing, Inc., Japan). The SPAD reading of each palm was taken at the lower, middle and upper portion of the leaf (2 mm × 3 mm) and averaged for each plant.

Proline content: 0.1 g fresh leaf sample was ground in 3% sulfosalicylic acid. The filtrate was boiled for 10 min and mixed with equal volumes of glacial acetic acid and 2.5% acidic ninhydrin. The mixture was boiled for 30 min and mixed with a double volume of toluene. The optical density of the upper aqueous phase was measured at 520 nm and proline concentration was determined from a standard curve. The proline content (g/g FW) was calculated as: (m1 × V1)/(m2 × V2) (m1, proline concentration based on the standard curve; V1, volume of the total extraction; m2, fresh weight of the sample; V2, volume of the test sample solution) (Liu et al., 2024).

Superoxide Dismutase (SOD) activity: 0.5 g fresh leaves were ground in 5 mL phosphate buffer. After centrifugation, the supernatant was collected and mixed with methionine solution, phosphate buffer, NBT solution, EDTA-Na_2_ solution, and riboflavin solution in light or dark for 30 min (phosphate solution as the control). The absorbance value was measured at 560 nm. SOD activity (U/g) as calculated as: 2(Ack-Ae) ×Vt/Ack×W×V0 (Ack, the absorbance value of the light control; Ae, the absorbance value of the sample; Vt (mL), the total volume of extract; W (g), the mass of the sample; V0 (mL), the sample volume).

### Protein characteristics

The signal peptide of expansin proteins was predicted using the online software SignalP (http://www.cbs.dtu.dk/services/SignalP/) (Armenteros et al., 2019). The structure domains of expansin proteins were determined through online software (http://smart.embl-heidelberg.de/) (Schultz et al., 2000). The sequence alignment among the expansins was conducted by the software DNAMAN and the evolutionary tree was constructed using MEGA 6 (Tamura et al., 2021). The online software ProtParam was used for analyzing the physicochemical properties of the proteins (https://web.expasy.org/protparam/) (Hoffer, 2011). The online software SOPMA (https://npsa-prabi.ibcp.fr/cgi-bin/npsa_automat.pl?page=/NPSA/npsasopma.html) was utilized to predict the secondary structure of the proteins (Deleage, 2017). The online software Swiss-model (http://swissmodel.expasy.org/) was applied for homology modeling construction of protein (Schwede et al., 2003). The software PyMOL was utilized to visualize the protein’s spatial structure.

## Results

### Characterisation of the expansin subfamily genes from Populus tomentosa

**Figure 1:**
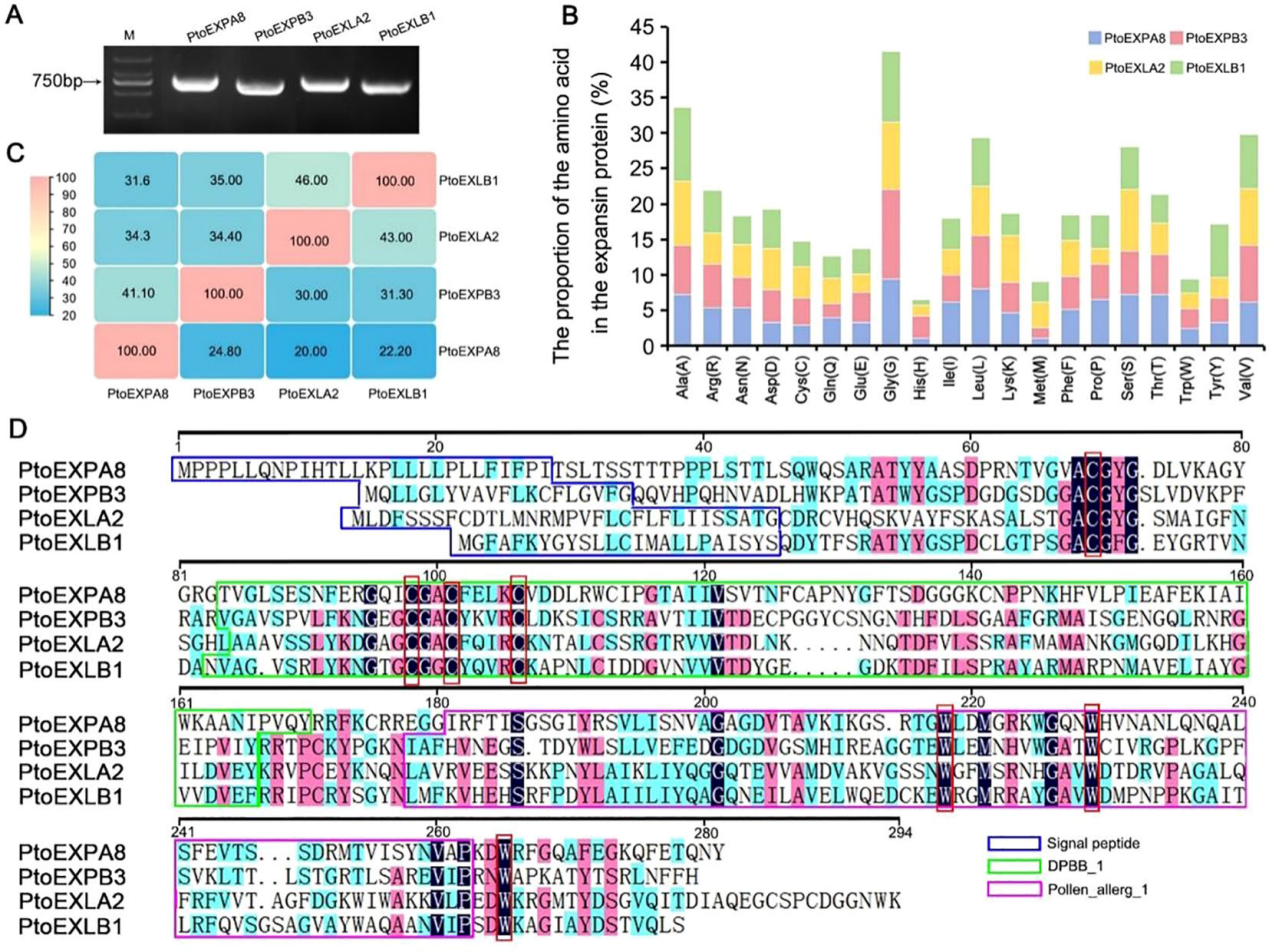
Sequence consistency of the expansin genes in *Populus tomentosa*. **(A)** PCR amplification of expansin genes in poplars; **(B)** proportion of the amino acids in the expansins (%); **(C)** sequence identity (%) between the two expansin genes in nucleotide (upper left) and amino acid (lower right); **(D)** amino acids alignment of the expansins.

The full-length fragments of the four expansin subfamily genes were amplified from the cDNA sample of *Populus tomentosa* TC1521 leaves using specific primers. Sequence alignment revealed that *PtoEXPA8, PtoEXPB3, PtoEXLA2*, and *PtoEXLB1* contained 831 bp, 789 bp, 825 bp, and 753 bp of nucleotides, encoding 276, 262, 274, and 250 amino acids, respectively. The four genes’ nucleotide identity ranged between 32.00% and 46.00%, while their amino acid identity ranged between 20.00% and 43.00% (**Figure 1C**). Several amino acids were found in different proportions in the four expansins: for example, methionine (M) ranged between 1.10% and 3.60% and histidine (H) ranged between 0.8% and 3.1%, while cysteine (C) and tryptophan (W), which play crucial roles in expansins’ functions, ranged between 2.90% to 4.40% and 2.00% to 2.70%, respectively (**Figure 1B**). Of them, the positional amino acids of 69C, 98C, 101C, 106C, 218W, 229W, and 265W were conserved in all the four expansins in a proportion of 36.84% of the total conserved amino acids (**Figure 1D**). Meanwhile, PtoEXLA2/PtoEXLB1 was found to have specific amino acids of 34C and 254W, PtoEXPA8/PtoEXPB3 the specific amino acids of 48W and 225W, and PtoEXPB3/PtoEXLA2/PtoEXLB1 the specific amino acids of 112C and 171C. Other amino acids were also present in the expansins, such as 53T in PtoEXPA8/PtoEXPB3/PtoEXLB1 and 100A in PtoEXPA8/PtoEXPB3/PtoEXLA2. These subfamily genes evidently had their own specific sequence characteristics.

**Figure 2:**
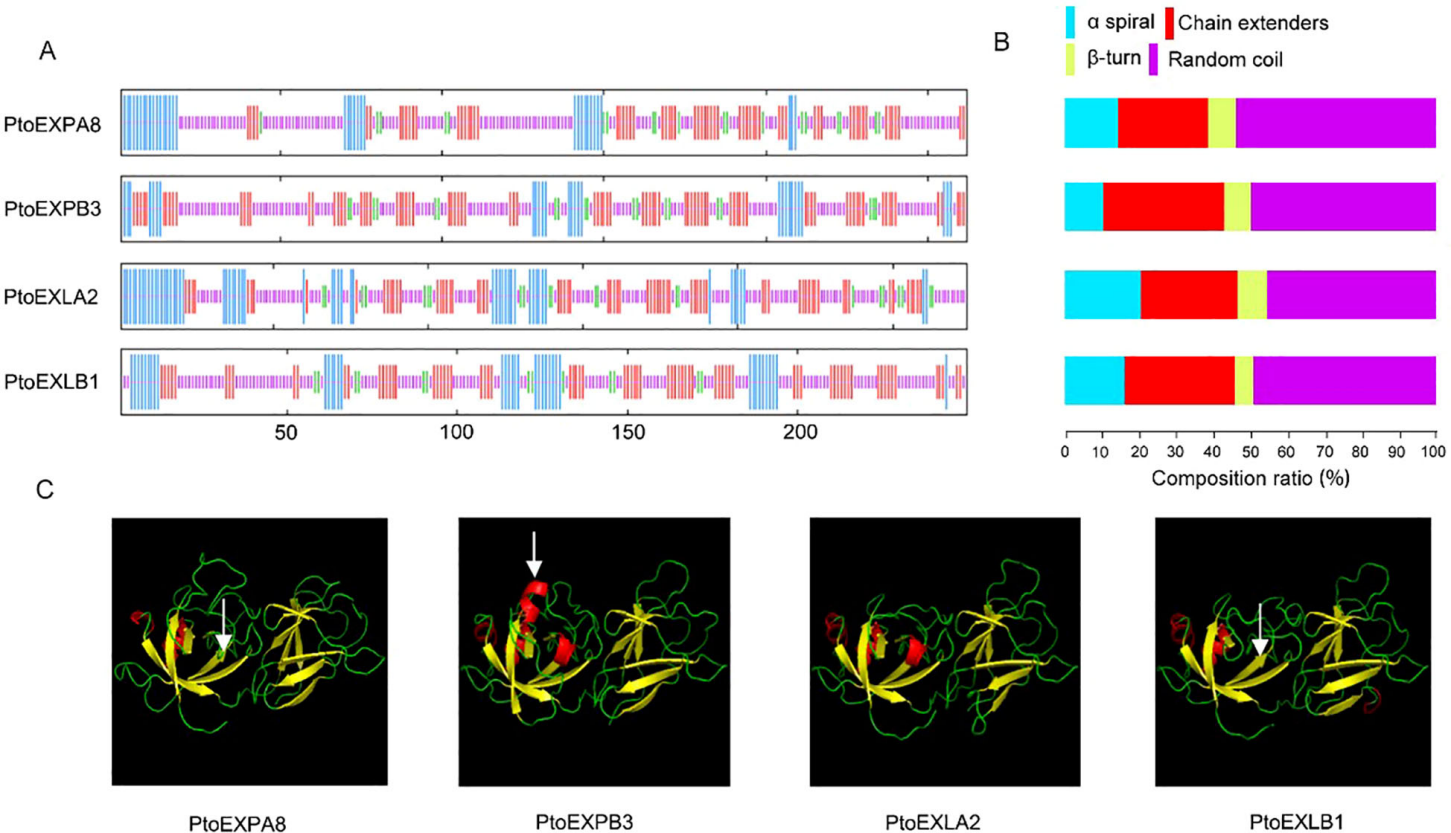
Structure of the four expansin proteins. **(A)** secondary structure; **(B)** proportion of the secondary components; **(C)** tertiary structure.

The four expansins were composed of three distinct domains: a signal peptide, a DPBB_1 catalytic domain, and a Pollen_allerg_1 binding domain (**Figures 1A, D**). Cysteine and tryptophan, the key amino acids, were mainly present in the catalytic domain and binding domain separately. Protein secondary structure prediction revealed that the all four proteins contained α-helices, extended strands, β-turns, and random coils in a similar weight. Of them, random coils were the most abundant, accounting for 49.33% of the total, followed by extended strands and α-helices accounting for 28.06% and 15.60%, and then finally by β-turns, accounting for 7.02% (**Figure 2A**). However, the proportion of these elements varied greatly in the four expansin proteins. For example, PtoEXPB3 had the least α-helices (10.69%) but PtoEXLA2 had the most (20.80%); PtoEXPA8 had the least extended strands (24.28%) but PtoEXPB3 had the most (32.44%); PtoEXLB1 had the least β-turns (5.2%) but PtoEXLA2 had the most (8.03%); and finally, PtoEXLA2 had the least random coils (45.26%) but PtoEXPA8 had the most (53.62%) (**Figure 2B**). Further, protein tertiary structure prediction revealed that the four expansins shared a similar framework. However, a few structures varied, which are shown using arrows in **Figure 2C**.

The physicochemical properties of the four expansins varied significantly: PtoEXPA8 was found to have the most amino acids (276) and the largest protein molecular weight (30.36 kDa), while PtoEXLB1 had the least (250) and the smallest (27.61 kDa); only PtoEXLB1 had a near-neutral isoelectric point (6.71), while the other three were all alkaline; PtoEXPA8 had the largest aliphatic index (80.22), indicating that it is thermophilic and adapts well to various environments, while the other three proteins were found to be sensitive. All four of them had an instability index value less than 40, indicating that they are stable, while PtoEXLA2 had the highest data of 34.98 and PtoEXPB3 had the lowest data of 24.41. Finally, they are all hydrophilic proteins (GRAVY value < 0), with PtoEXPB3 being the lowest (-0.17) and PtoEXLA2 the highest (-0.068). (**Table 1**).

**Table 1:** Physicochemical property of the poplar expansins.

Protein	Number of amino acids	Molecular weight (kDa)	Isoelectric point	Aliphatic index	Instability Index	GRAVY
PtoEXPA8	276	30.36	9.3	80.22	33.48	-0.133
PtoEXPB3	262	28.63	8.51	74.77	24.41	-0.17
PtoEXLA2	274	29.90	8.78	73.69	34.98	-0.068
PtoEXLB1	250	27.61	6.71	76.12	30.51	-0.097

### The expression patterns of the four expansin genes in *Populus tomentosa*

**Figure 3:**
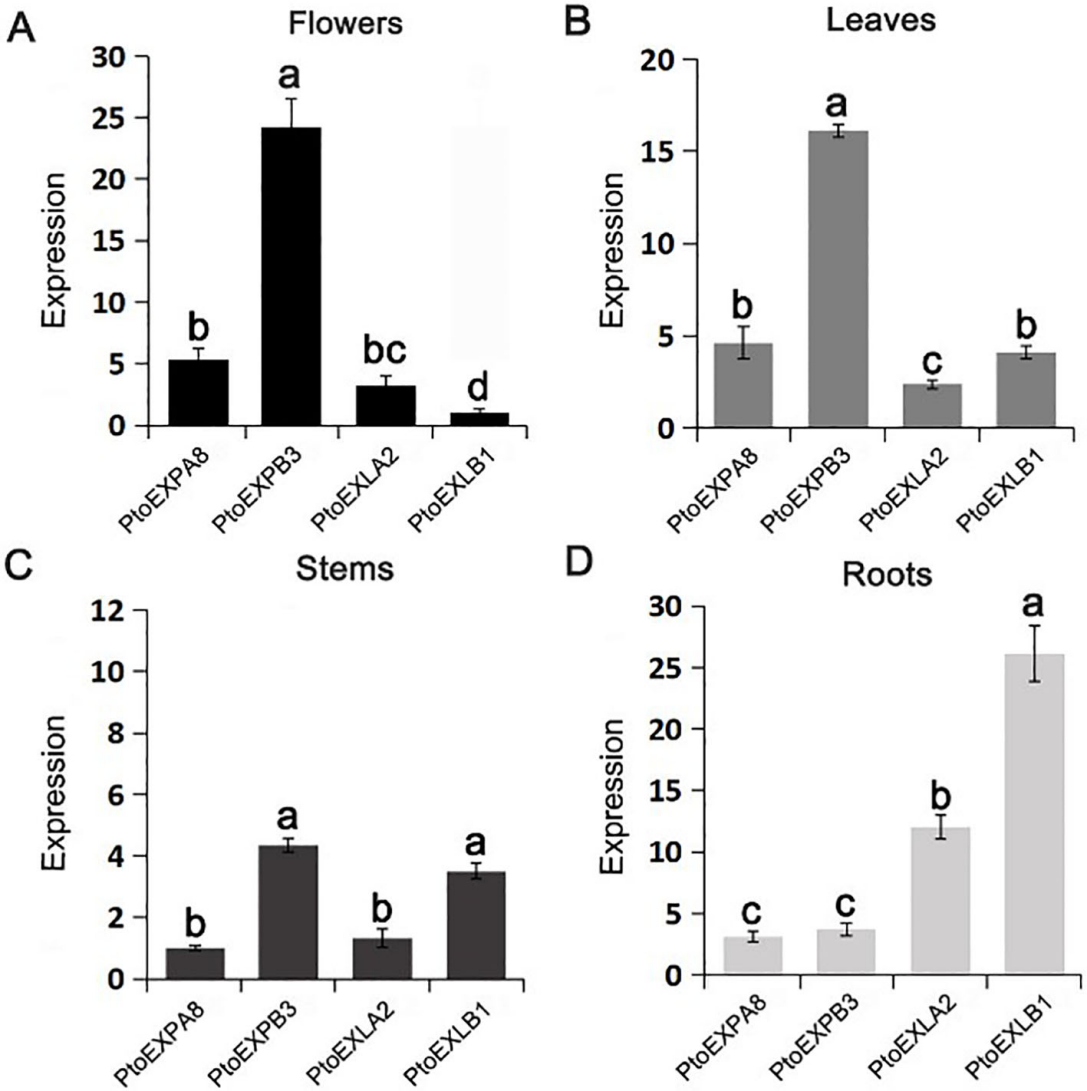
Expression patterns of the expansin genes in poplar tissues and organs. **(A)** flowers; **(B)** leaves; **(C)** stems; **(D)** roots. The values are mean of the expression amount of the expansin genes ± SD, n=3. The different letters denote significant differences among the genes by LSD multiple comparison test.

The RT-PCR results revealed that the four expansin genes were expressed in poplar tissues and organs quite differently. *PtoEXPB3* exhibited the highest expression levels in flowers, which were 4.35, 7.20, and 23.12 folds higher than those of *PtoEXPA8, PtoEXLA2*, and *PtoEXLB1*, respectively; it also exhibited the highest expression levels in leaves, which were 3.50, 6.66, and 3.92 folds higher than those of *PtoEXPA8*, *PtoEXLA2*, and *PtoEXLB1* (**Figures 3A, B**). In stems, *PtoEXPB3* and *PtoEXLB1* demonstrated relatively high expression levels, which were 4.26 and 3.29 folds higher than those of *PtoEXPA8* and 3.43 and 2.65 folds higher than those of *PtoEXLA2* (**Figure 3C**). In roots, *PtoEXLB1* had the highest expression level, which was 8.38, 7.05, and 2.17 folds higher than those of *PtoEXLA2, PtoEXPB3*, and *PtoEXPA8* (**Figure 3D**). These results indicate that the expansin subfamily genes play different roles in plant growth and development.

**Figure 4:**
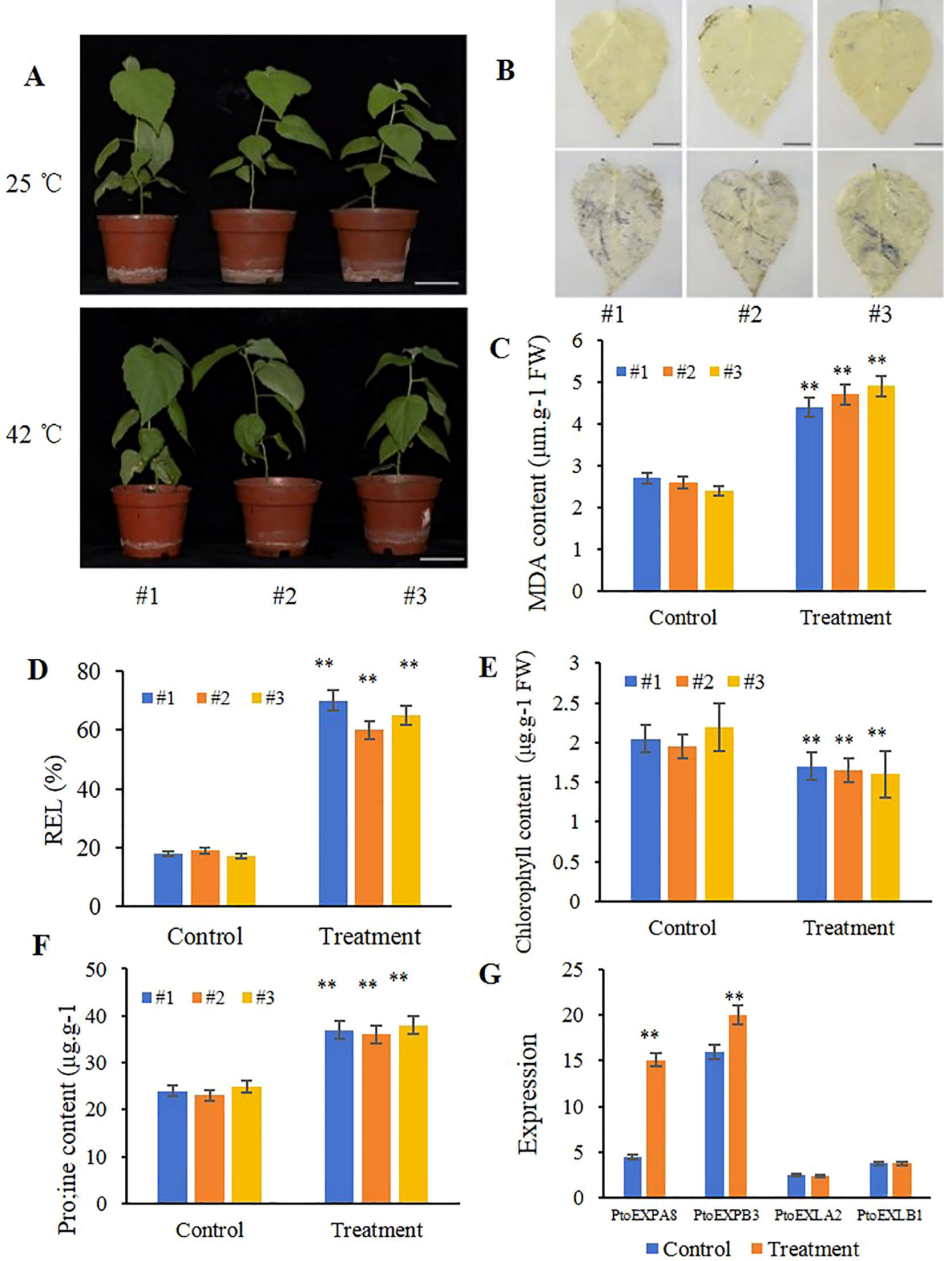
Performance of *Populus tomentosa* under 42 °C stress for 3 d and expression of the expansin genes. **(A)** morphological performance of three-month-old poplar plants (Bars=6cm); **(B)** NBT staining of the poplar leaves (Bars=1cm); **(C)** MDA content; **(D)** relative electrical leakage (REL); **(E)** chlorophyll content; **(F)** proline content. **(G)** gene expression in leaves under treatments by RT-PCR. The values are mean of three replicates ± SD. **indicates significant difference between the control (25°C) and heat treatment (42°C) (P<0.01), determined by student’s t-test. #1, #2 and #3 are poplar clones.

When treated in 42 °C for 3 days, the poplar leaves drooped and wilted a little (**Figure 4A**). NBT staining revealed that the leaves were really intensified (**Figure 4B**). And, the MDA content in the leaves increased by 88.07% compared to that control [(heat stress–control)/control] (**Figure 4C**), the REL increased by 317.84% (**Figure 4D**), the chlorophyll content decreased by 19.70% (**Figure 4E**), and the proline content increased by 49.40% (**Figure 4F**). These results indicate that high temperature caused the poplar leaves with a certain damage.

*PtoEXPA8* and *PtoEXPB3*, the expression assays revealed, were significantly upregulated under high temperature stress, increasing by 232.61% and 25.00%, respectively, compared to the control. Both were accordingly involved in the plant resistance process. In contrast, the expression levels of *PtoEXLA2* and *PtoEXLB1* did not change significantly during heat stress (**Figure 4G**).

### The effects of the poplar expansin genes on plant growth and development

**Figure 5:**
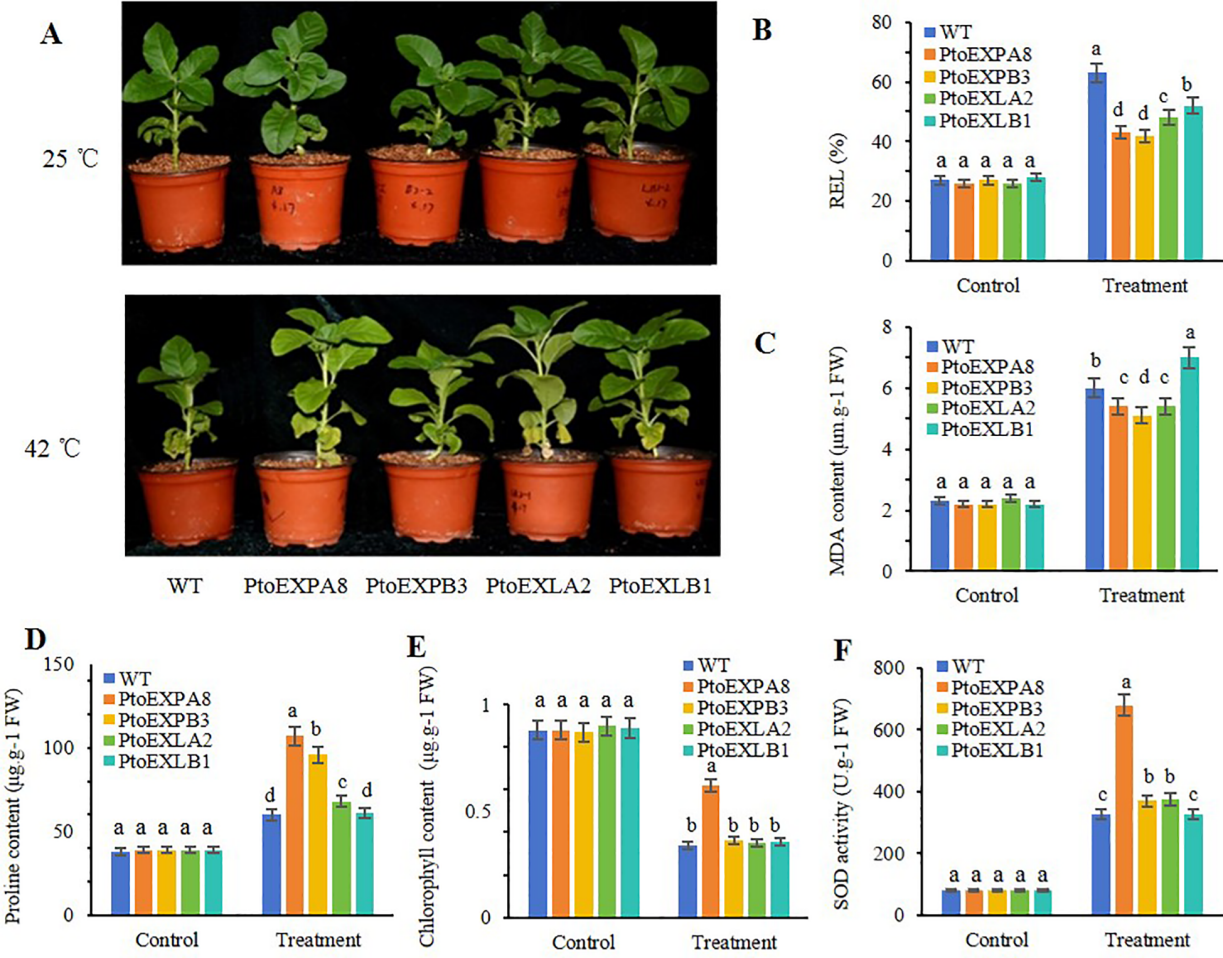
Growth and development performance of the transgenic tobacco plants expressing poplar expansin genes. **(A)** expression of the poplar expansin genes in tobacco plants (*UBQ* as the reference gene); **(B)** plant height; **(C)** flower diameter; **(D)** flowering time; **(E)** leaf area, leaf length, leaf width and length/width ratio; **(F)** 1000-seeds weight. The values are mean of three replicates ± SD. The different letters denote statistically significant differences via LSD multiple comparison test.

The four expansin genes were individually introduced into tobacco plants. PCR assays showed them expressed in the plants similarly (**Figure 5A**). The measurements of the plant growth and development traits revealed different contributions of the four poplar expansin genes. Compared to the wild type (WT), the transgenic tobacco lines of *PtoEXLA2* and *PtoEXLB1* grew faster, with plant height increasing by 6.14% and 5.78%, respectively [(transgenic line – WT)/WT]. In contrast, the transgenic lines of *PtoEXPA8* and *PtoEXPB3* were similar as that of the WT (**Figure 5A**). Further, the flower size measurement revealed that the *PtoEXPB3* transgenic plants had a diameter of 2.34 cm, 7.8% longer than that of WT. The *PtoEXLB1* and *PtoEXLA2* transgenic lines reduced the flower diameter by 3.69% and 4.15%, respectively, compared to the WT, and no significant difference was present between the *PtoEXPA8* transgenic plants and the WT (**Figure 5B**). The average flowering time of the *PtoEXPB3* transgenic line was 21 days earlier than that of the WT (**Figure 5C**), and the transgenic lines of *PtoEXPA8*, *PtoEXLB1*, and *PtoEXLA2* had similar flowering times as the WT. Moreover, the leaf growth of the transgenic plants and WT varied significantly. The ninth leaf length of the *PtoEXPA8* and *PtoEXPB3* transgenic plants were longer than that of the WT, with an increase of 16.74% and 15.82%, while no significant changes were found between the *PtoEXLA2* and *PtoEXLB1* transgenic plants and WT. The ninth leaf width of the *PtoEXPA8*, *PtoEXPB3*, and *PtoEXLA2* transgenic plants increased by 20.18%, 23.39%, and 23.68%, respectively, compared to the WT, while no significant changes were found between the *PtoEXLB1* transgenic plants and WT. Calculating the leaf length/width ratio revealed that the *PtoEXPB3* and *PtoEXLA2* transgenic lines had a reduction of 6.13% and 15.26% compared to the WT, while no significant changes were found between the *PtoEXPA8* and *PtoEXLB1* transgenic lines and the WT. Moreover, the leaf area of the *PtoEXPA8* and *PtoEXPB3* transgenic plants increased by 29.50% and 27.05%, while it was reduced in the *PtoEXLB1* transgenic plants by 13.58%. No significant change occurred in the *PtoEXLA2* transgenic line compared to the WT (**Figure 5D**). Further, the *PtoEXLA2* transgenic line had a 1000-seed weight of 19.7 mg, with a decrease of 44.82% compared to WT. In contrast, the *PtoEXPB3* transgenic line demonstrated an increase of 14.85%, and no significant differences were found between the *PtoEXPA8* and *PtoEXLB1* transgenic lines and WT (**Figure 5E**).

### Contribution of the poplar expansin genes to heat resistance

**Figure 6:**
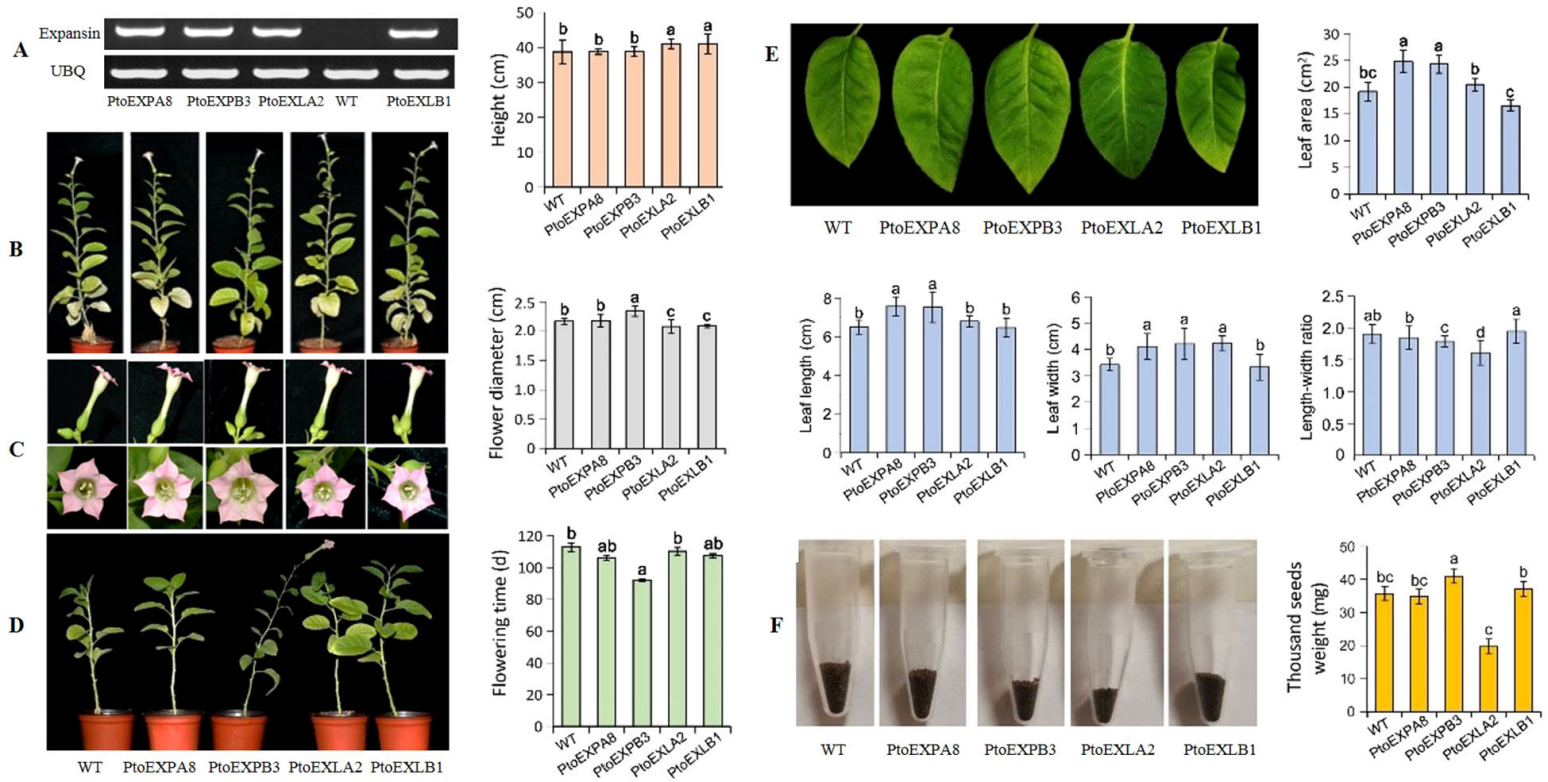
Performance of the transgenic tobacco plants expressing poplar expansin genes under heat stress. **(A)** morphological performance; **(B)** REL; **(C)** MDA content; **(D)** proline content; **(E)** chlorophyll content; **(F)** SOD activity. The values are mean of three replicated ± SD. The different letters denote statistically significant differences via LSD multiple comparison test.

Owing to high temperature stress, the tobacco leaves wilted, and the plants’ mechanical strength decreased significantly. Comparably, the transgenic lines of *PtoEXPA8, PtoEXPB3*, and *PtoEXLA2* maintained better performance than that of the WT, while the transgenic line of *PtoEXLB1* performed similarly as the WT (**Figure 6A**).

Further, the REL and MDA content value of the tobacco lines stress increased when subjected to high temperature stress, while the chlorophyll content, proline content, and SOD activity decreased. The transgenic lines of the four expansin genes performed differently. For example, the transgenic lines of *PtoEXPA8, PtoEXPB3, PtoEXLA2*, and *PtoEXLB1* had lower REL (0.68, 0.66, 0.76, and 0.83 folds of the WT) (transgenic line/WT) and lower MDA content (0.85, 0.81, 0.85, and 1.09 folds of the WT), respectively (**Figures 6B, C**). They also had high chlorophyll content (1.84, 1.08, 1.05, and 1.07 folds of the WT) (**Figure 6D**), high proline content (1.76, 1.59, 1.13, and 1.03 folds of the WT) (**Figure 6E**), and high SOD activity (2.11, 1.16, 1.17, and 1.00 folds of the WT) (**Figure 6F**). Evidently, the four genes inconsistently advanced the physiological conditions under heat stress.

A principal component analysis was conducted on the five assayed physiological indicators. The cumulative contribution rate of principal factors 1 and 2 was 86.07%, which could effectively summarise the information of most of the original data. The contribution rate of principal factor 1 reached 71.99%. The date of all the five physiological indicators were significantly correlated with principal factor 1. Among them, the coefficients of proline content, SOD activity, and chlorophyll content were positively correlated, being 0.92, 0.87, and 0.86, respectively. The coefficients of REL and MDA content were negatively correlated, with values of -0.83 and -0.76, respectively. The contribution rate of principal factor 2 was 14.07%, and the coefficients of each indicator also varied (**Table 2**), indicating that the subfamily expansin genes contributed to the physiological index improvement differently. Further, the total score for *PtoEXPA8* was the highest at 2.75, followed by *PtoEXPB3, PtoEXLA2, PtoEXLB1*, and WT (**Table 3**). All the results indicated that the subfamily expansin genes contributed unequally to the plant resistance against heat stress, and *PtoEXPA8* was the most resistant gene.

**Table 2:** Principal component coefficients and contribution rate of the physiological indexes.

Index	Principal factor 1	Principal factor 2
SOD activity	0.87	0.40
MDA content	-0.76	0.54
Proline content	0.92	-0.04
Chlorophyll content	0.86	0.37
REL	-0.83	0.34
Contribution rate (%)	71.99	14.07
Cumulative contributions (%)	71.99	86.07

**Table 3:** Principal component and comprehensive scores of the expansin gene.

Plants	Principal factor 1 score	Principal factor 2 score	Total score*	Ranking
WT	-1.59	-0.20	-1.36	5
PtoEXPA8	3.58	-1.49	2.75	1
PtoEXPB3	1.36	1.07	1.31	2
PtoEXLA2	0.23	1.03	0.36	3
PtoEXLB1	-1.04	-0.08	-0.88	4
Weight*	0.84	0.16		

*Weight means the ratio of the variance contribution rate of each principal component to the total principal components. Total score means the sum of the principal component scores.

## Discussions

In the process of evolution, expansin genes underwent serious sequence variations, and they have been differentiated into four subfamilies: EXPA, EXPB, EXLA, and EXLB. A sequence alignment revealed that the amino acid identity between the subfamily genes only ranged between 33.7% and 50.4%, comparably 48.1%-94.2% among the same subfamily genes (Kende et al., 2004; Li et al., 2014). These sequence variations probably resulted in the expansin subfamily having a function bias in plant growth and stress resistance.

### Diversity of expansin genes functioning in plant growth and development

Some reports have shown that expansins are widely involved in plant growth and development. For example, the transgenic *Arabidopsis* plants of *VvEXPA14* and *VvEXPA18* increases the rosette leaf size (Suzuki et al., 2015). Both *ClEXPA1* and *ClEXPA2* from spruce thicken the xylem cell walls (Wang et al., 2011). The *TaEXPA2* overexpression line increases seed yield and pod number, while the *TaEXPB23* overexpression produces larger leaves and longer internodes (Chen et al., 2016; Xing et al., 2009). The transgenic tobacco plants of *PtEXLA1* feature a larger corolla than the WT (Liu et al., 2024). *OsEXPA17* and *OsEXPB2* are involved in root hair formation (Zou et al., 2015; Yu et al., 2011), and *OsEXPA8* participates in root development (Ma et al., 2013). Moreover, *ZmEXPB15* is involved in regulating grain size and weight, while *ZmEXPA5* reduces the anthesis-silking interval and improves grain yield (Sun et al., 2022a; Tao et al., 2022). In this study, the following results were found: the overexpression of *PtoEXPA8* increased the plant leaf area, *PtoEXLA2* and *PtoEXLB1* increased the plant height, and *PtoEXPB3* influenced flowering, flower size, leaf area, and 1000-grains weight (**Figure 6**). Seemly, the EXPA subfamily was more likely involved in vegetative growth, while the EXPB subfamily was more associated with reproductive growth (Chen et al., 2021). The most convincing correlation between morphological reduction and expansin clades loss provided more evidence for this inference, including genes belonging to *EXPA-I* (leaf development and abscission) (Cho and Cosgrove, 2000), *EXPA-X* (root hair development) (Yu et al., 2011), *EXPA-VI* (lateral root development) (Lee and Kim, 2013), and *EXPB-I* (pollen tube development) (Valdivia et al., 2009).

### Diversity of expansin genes functioning in resistance to adversity

Some expansin genes were involved in plant stress resistance. For example, the survival rates of the transgenic *Arabidopsis* plants with *TaEXPA19-A* or *TaEXPA19-D* were 77.3% and 73%, respectively, compared to the WT (63%) under low-temperature stress (Li et al., 2024). The expression of *OfEXLA1* in *Arabidopsis* improved the plant survival rate under salt stress conditions, with a value of 46.3% compared to the 15.7% of the WT (Dong et al., 2024). In drought stress conditions, the chlorophyll content of the *PtEXLA1* transgenic tobacco plants increased by 36.67% compared to the WT, while the REL and MDA content decreased by 20.00% and 23.33%, respectively (Liu et al., 2024). In heavy metal stress conditions, the activities of H-ATPase, V-ATPase, and PPase were higher in the transgenic lines of *TaEXPA2* than those in the WT, which facilitated the transport of cadmium (Cd) into vacuoles and increased the plants’ resistance to heavy metal stress (Ren et al., 2018). In salt stress conditions, the REL and MDA content of the *SmEXPA13* transgenic tobacco were 19.57% and 26.92% lower than those of the WT, respectively, which significantly improved the performance of the plants under stress (Zhang et al., 2023). The expansin genes thus mediated in different abiotic stress resistance and exhibited differences in doing so.

Furthermore, the resistance level of the expansin genes to same stress was also different. For example, when subjected to heat stress, the soluble sugar content of the transgenic tobacco lines of *AstEXPA1* and *PtoEXPA8* increased by 36.13% and 41.65%, respectively, compared to the WT (Zhang et al., 2017, 2019). Moreover, under heat stress, the REL and MDA content of the *PtEXLA1* transgenic tobacco plants decreased by 20.00% and 16.67% compared to the WT, respectively (Liu et al., 2024), whereas the transgenic plants of *PtoEXPA8* demonstrated a 1.17-fold lower REL value and a 1.22-fold lower MDA content than that of WT plants (Zhang et al., 2019). In this study, the REL of the transgenic lines *PtoEXPA8, PtoEXPB3, PtoEXLA2*, and *PtoEXLB1* were 0.68, 0.66, 0.76, and 0.83 folds of the WT (transgenic line/WT), and the MDA content was 0.85, 0.81, 0.85, and 1.09 folds of the WT, respectively. These results somehow demonstrated the gene specificity and even subfamily or classification attributes in functioning. Several clades also contributed to abiotic stress responses, including *EXPA-III* (Yan et al., 2014), *EXPB-I* (Geilfus et al., 2015), and *EXLA-I* (Abuqamar et al., 2013), which further supported this hypothesis.

### Sequence variation of expansins responding to their function differentiation

Amino acid mutations in proteins, particularly those in structural domains, can severely impact protein expression and function. For example, cysteine (C) was recognized as a key amino acid in expansins. When all the cysteine residues in PpEXP1 from *Prunus persica* were substituted with serine, the secreted expansin of the transformed yeast with normal PpEXP1 and the mutant were 0.58 mg·L-1 and 4.3 mg·L-1, respectively. Cysteine could thus greatly affect the heterologous expression of the expansin gene (Matsuyama et al., 2020).

Tryptophan (W) is another critical amino acid in expansins. When the 211W in tomato expansin SlExp1 was mutated to serine (S), the mutant fruits were harder by 41% compared to the control (Bhat et al., 2010). The mutation was inferred to have seriously affected the binding of the expansin to cellulose. Another case also revealed the importance of tryptophan residues in protein function. The five conserved tryptophan residues in the cellulose-binding domain of xylanase A from *Pseudomonas fluorescens* were changed to alanine (A) and phenylalanine (P), which significantly reduced the binding ability of the protein to cellulose. Of them, 13W, 34W, and 38W were found to be crucial for maintaining the cellulose-binding capacity of the domain (Poole et al., 1993). In this study, the proportions of C and W in the four expansins ranged between 2.90% and 4.40% and 2.00% and 2.70% (**Figure 3B**). Several C and W were greatly conserved, such as 69C, 98C, 101C, 106C, 218W, 229W, and 265W, which accounted for a large proportion of the total identical amino acids of the four expansins (36.84%) and were necessary for the expansins to perform their basal function. However, several C and W were specific in the expansins, such as 111C and 170C for PtoEXPB3/PtoEXLA2/PtoEXLB1 and 223W for PtoEXPA8/PtoEXPB3. These probably contributed to their diverse functions.

Amino acid mutations could also affect the secondary structure of proteins and further influence their performance. The secondary structure of proteins is primarily composed of α-helices, extended strands, β-turns, and random coils. One study investigated the role of α-helix against freeze stress, in which a modified antifreeze protein (AFP) with 20% more fraction helix effectively inhibited ice crystal growth at a concentration of 0.025 mM, while the normal AFP required a higher concentration (0.061 mM–0.198 mM). These results indicated a direct correlation between α-helix content and antifreeze activity (Chakrabartty and Hew, 1991). The four expansins in the present study demonstrated significant variations in their secondary structure elements: PtoEXPB3 and PtoEXLA2 exhibited α-helix proportions of 10.69% and 20.80%, and PtoEXLA2 and PtoEXLB1 exhibited β-turn proportions of 8.03% and 5.20%, respectively (**Figure 4**). These variations, we inferred, significantly affected their functions. More studies are necessary to explore their mechanisms in depth.

## Data Availability

The original contributions presented in the study are included in the article/**Supplementary Material**. Further inquiries can be directed to the corresponding author.
