# Supplementary material for: Function diversity of the expansin subfamily genes in Populus tomentosa
Source: Front Plant Sci. 2026 Jan 22;16:1741986. doi: 10.3389/fpls.2025.1741986 (PMC12872768; doi:10.3389/fpls.2025.1741986)
Supplement: Supplementary file 1 [file Table1.docx]

Supporting Information

Table S1. The specific primers of four poplar expansin genes used for expression test

| Gene | Upward（5’-3’） | Backward（5’-3’） |
| --- | --- | --- |
| *PtrEXPA8* | AAGTGTGTTGATGATCTGAG | ATCTTCACTGCAGTGACATC |
| *PtrEXPB3* | GTACGGGTCATTAGTGGATG | TTCCACTAGAAGGGATAGCC |
| *PtrEXLA2* | CTCTGCTACTGGTTGTGATCG | TCAAGACGTCTTGACCCATGC |
| *PtrEXLB1* | CTGACTTCATCCTCAGCCCAC | CTGCCACTTACTTGGAACCTC |
| *UBQ* | CCAAGCCCAAGAAGATCAAGC | GCACCGCACTCAGCATTAGG |

Table S2. The specific primers of four poplar expansin genes used for cloning

| Gene | Upward（5’-3’）* | Backward（5’-3’） |
| --- | --- | --- |
| *PtrEXPA8* | CCCAAGCTTATGCCACCACCATTACCCC | TGCTCTAGATTAAGTCTCAAATTGCTTGCCTTC |
| *PtrEXPB3* | CCCAAGCTTATGCAGCTCTTGGGGTTAC | TGCTCTAGATTAATGGAAGAAATTGAGCCTAGAGG |
| *PtrEXLA2* | CCCAAGCTTATGCTTGATTTCTCTTCTTCC | TGCTCTAGATCATTTCCAAATCCCATCAC |
| *PtrEXLB1* | CCCAAGCTTATGGGATTTGCATTTAAATATGGC | TGCTCTAGATTAAGAAAGCTGAACGGTAG |

*The restriction sites for HindIII and XbaI were underlined for upward and backward primers, respectively.
